# The Milk Active Ingredient, 2′-Fucosyllactose, Inhibits Inflammation and Promotes MUC2 Secretion in LS174T Goblet Cells In Vitro

**DOI:** 10.3390/foods12010186

**Published:** 2023-01-01

**Authors:** Qianqian Yao, Huiying Li, Yanan Gao, Nan Zheng, Véronique Delcenserie, Jiaqi Wang

**Affiliations:** 1 State Key Laboratory of Animal Nutrition, Institute of Animal Sciences, Chinese Academy of Agricultural Sciences, Beijing 100193, China; 2 Department of Food Science, Faculty of Veterinary Medicine, FARAH, University of Liège, 4000 Liège, Belgium; 3 Beijing Key Laboratory of Food Processing and Safety in Forestry, College of Biological Sciences and Technology, Beijing Forestry University, Beijing 100085, China

**Keywords:** 2′-fucosyllactose (2′-FL), goblet cell, MUC2, NLRP6

## Abstract

In several mice inflammatory models, human milk oligosaccharides (HMOs) were shown to protect the intestinal barrier by promoting mucin secretion and suppressing inflammation. However, the functions of the individual HMOs in enhancing mucin expression in vivo have not been compared, and the related mechanisms are not yet to be clarified. In this study, we investigated the modulatory effects of 2′-fucosyllactose (2′-FL), 3′-sialyllactose (3′-SL), galacto-oligosaccharide (GOS) and lactose (Lac) on goblet cells’ functions in vitro. The appropriate dosage of the four chemicals was assessed in LS174T cells using the CCK-8 method. Then they were supplemented into a homeostasis and inflammatory environment to further investigate their effects under different conditions. Mucin secretion-related genes, including mucin 2 (*MUC2*), trefoil factor family 3 (*TFF3*), resistin-like β (*RETNLB*), carbohydrate sulfotransferase 5 (*CHST5*) and galactose-3-O-sulfotransferase 2 (*GAL3ST2*), in LS174T cells were detected using quantitative RT-qPCR. The results showed that 2′-FL (2.5 mg/mL, 72 h) was unable to increase MUC2 secretion in a steady-state condition. Comparatively, it exhibited a greater ability to improve mucin secretion under an inflammatory condition compared with GOS, demonstrated by a significant increase in *TFF3* and *CHST5* mRNA expression levels (*p* > 0.05). However, 3′-SL and Lac exhibited no effects on mucin secretion. To further investigate the underlying mechanism via which 2′-FL enhanced goblet cells’ secretion function, the NOD-like receptor family pyrin domain containing 6 (*NLRP6*) gene, which is closely related to MUC2 secretion, was silenced using the siRNA method. After silencing the *NLRP6* gene, the mRNA expression levels of *MUC2, TFF3* and *CHST5* in the (2′-FL + tumor necrosis factor α (TNF-α) + NLRP6 siRNA) group were significantly decreased compared with the (2′-FL + TNF-α) group (*p* > 0.05), indicating that NLRP6 was essential for MUC2 expression in goblet cells. We further found that 2′-FL could significantly decrease toll-like receptor 4 (TLR4, *p* < 0.05), myeloid differential protein-88 (MyD88, *p* < 0.05) and nuclear factor kappa-B (NF-κB, *p* < 0.05) levels in LS174T inflammatory cells, even when the NLRP6 was silenced. Altogether, these results indicated that in goblet cells, 2′-FL exerts its function via multiple processes, i.e., by promoting mucin secretion through NLRP6 and suppressing inflammation by inhibiting the TLR4/MyD88/NF-κB pathway.

## 1. Introduction

Human milk oligosaccharides (HMOs) are an important component of breast milk, which are mainly fermented in the large intestine [1]. They promote immune system maturation by supporting the formation of a milk-oriented microbiota [2] and strengthens the epithelial barrier [3]. HMOs are generally based on a lactose molecule and elongated into straight or branched chain by different numbers of Galβ1-3GlcNAc or Galβ1-4GlcNAc units. Sialic acid and fucose are extended on the main chain as a modified group. Over 200 structures of HMOs have been identified, of which about 162 have been structurally characterized [4]. One of these structures, 2′-fucosyllactose (2′-FL), is the most abundant fucosylated HMO in breast milk, accounting for about 25% of HMOs, while 3′-sialyllactose (3′-SL) is the main HMO acid, comprising about 6% of all HMOs. Although HMOs were reported to improve the mucin barrier in several mice inflammatory models [5,6], it remains unclear whether this is microbiota-dependent, or due to HMOs or a fraction of it. Moreover, the difference in improving mucin secretion between different modified HMOs has not yet been elucidated.

In some cases, the infant formulas are commonly used when breastfeeding is unavailable [7]. Due to technical challenges in obtaining oligosaccharides from human milk, galacto-oligosaccharides (GOS) are added to infant formula to mimic the function of HMOs. Though GOS have been reported to enhance mucin expression [8], the structural differences between them make it doubtful whether GOS exert the same functions as HMOs. In addition, since lactose (Lac) is the most abundant sugar carbohydrate in dairy products, and it is also the core structure of oligosaccharides and GOS, we investigated the effects of Lac on mucin secretion.

The mucus layer acts as a barrier to segregate microorganisms from the colonic epithelia. MUC2, the main protein in the mucus layer, is primarily produced by goblet cells. It forms a single nonmoving mucus layer that tightly adheres to colonic epithelia [9,10]. Several genes are involved in the mucin secretion process and regulation of MUC2 production. Trefoil factor 3 (*TFF3*) is co-secreted and synergizes with MUC2 to maintain the integrity of the mucin barrier [11]. Another two related products, Golgi sulfotransferases galactose-3-O-sulfotransferase 2 (*GAL3ST2*) and carbohydrate sulfotransferase 5 (*CHST5*), are essential sulfur transferases located in the Golgi apparatus. It was found that adding sulfonyl groups to mucin improved the function of the mucus barrier and protected against adherence of pathogens [12]. The resistin-like molecule beta (*RETNLβ*) drives spontaneous colitis in Muc2-deficient mice by promoting commensal microbial dysbiosis [13].

NOD-like receptor family pyrin domain containing 6 (*NLRP6*) is expressed in goblet cells and participates in the regulation of the NF-κB signaling pathway [14]. NLRP6^−/−^ mice were shown to exhibit dysfunctional mucus granule exocytosis [14], indicating the involvement of *NLRP6* in the mucin secretion process. In our previous study [15], NLRP6 was found to be involved in the process of 2′-FL alleviating DSS-induced colitis and promoting MUC2 expression in C57BL/6J mice. Thus, in this study, we further explore the link between NLRP6, mucus secretion, and HMOs in goblet cells (LS174T cells).

In our present study, we compared the potential of 2′-FL, 3′-SL, GOS and lactose to improve mucin secretion under a steady-state condition and an inflammatory setting by testing for the expression of MUC2 secretion-related genes. NLRP6 siRNA was also used to confirm the findings.

## 2. Materials and Methods

### 2.1. Chemicals

2′-fucosyllactose (2′-FL, cat# GY1141, ≥98%) and 3′-Sialyllactose sodium salt (3′-SL, cat# GY1143, ≥98%) were purchased from HuicH Biotech Co., Ltd. (Shanghai, China). Galacto-oligosaccharide (GOS, cat#G9150, ≥70%) and lactose (Lac, cat#SL8740, ≥98%) were purchased from Solarbio Life Sciences (Beijing, China). LS174T cells (cat#CL-0145) were purchased from Procell Life Science & Technology Co., Ltd. (Wuhan, China). Recombinant human tumor necrosis factor α (TNF-α, cat#200-13-2) was obtained from Peprotech (Rocky Hill, NJ, USA). Anti-MUC2 Polyclonal Antibody (cat#K106881P) and FITC Goat Anti-Rabbit IgG (cat#A22120) were purchased from Bioss (Beijing, China). Anti-NLRP6 Antibody (cat#ABF29) was obtained from Merch (Shanghai, China). The siRNA of NLRP6 was designed and produced from GenePharma (Shanghai, China). Lipofectamine™ 3000 transfection reagent (cat#L3000008) was purchased from ThermoFisher Scientific (Shanghai, China).

### 2.2. Cell Culture

LS174T cells (human intestinal goblet cells) were grown using complete growth medium (MEM + 10% FBS + 1% *P*/*S*) at 37 °C in a cell incubator with 5% CO_2_. The cells were plated into 6-well plates (5 × 10^5^ cells/mL medium) until they reached 70–80% adherence, then the old medium was replaced with 1 mL of fresh medium containing different concentrations of the. four chemicals (0, 1.0, 1.5, 2.0, 2.5, 3.0 and 5.0 mg/mL of 2′-FL or 3′-SL; 0, 5, 7.5, 10, 12.5, 15 and 20 mg/mL of GOS or Lac). The selection of 2′-FL and 3′-SL dosages were based on the work of Figueroa-Lozano et al. [16] and He et al. [17], while those of GOS and Lac were based on studies by Ghosh et al. [8]. The cells were allowed to grow for another 24, 48 and 72 h, followed by a cell viability assessment using CCK-8 to identify the proper dosage and culture time.

### 2.3. Cellular Inflammatory Model Construction by TNF-α Treatment

LS174T cells were seeded into 12-well plates and cultured to 70–80% adherence. The proper dosage of 2′-FL, 3′-SL, GOS and Lac was applied for 48 h and then the medium was replaced with new medium containing TNF-α (10 ng/mL) [18]. At the end of the stimulation, LS174T cells were homogenized with TRIzol reagent and stored at −20 °C for RNA extraction.

### 2.4. Animal Model

A total of 18 C57BL/6J male mice (18–22 g) were divided into three groups: the control group, the DSS group, and the DSS + 2′-FL group, as previously described (permission number of ethical file: IAS-2021-03) [15]. Mice in the DSS + 2′-FL groups received 0.3 mL of 2′-FL at 400 mg/kg b.w. orally once a day from day 0 to 21. An equal amount of PBS was simultaneously given to the mice in the control and DSS groups at the same time. To create a colitis model, mice in the DSS group were given 5% (*w*/*v*) DSS *ad libitum* in their water for 7 consecutive days, between day 14 and day 21. At the end of the experiment, immunofluorescence staining for MUC2 protein and PAS staining in the colon tissue were performed.

### 2.5. siRNA Gene Treatment

LS174T cells were seeded into 6-well plates and cultured until they adhered to the wall. The medium was replaced by an FBS-free and A/P-free medium containing siRNA fragments of *NLRP6* (designed by GenePharma) and then cells were continuously cultured for 24 h. Next, 2.5 mg/mL 2′-FL was added for 24 h followed by a TNF-α (10 ng/mL) challenge for 48 h. At the end of the experiment, 1 mL of TRIzol reagent was added into the wells and cells were harvested and stored at −20 °C for RNA extraction. The primers of *NLRP6* siRNA are shown in Table 1.

### 2.6. Cell Immunofluorescence Staining

LS174T cells were fixed in 4% (vol./vol.) paraformaldehyde for 15 min, followed by permeabilization with Triton-X-100 (2%, vol./vol.) for 10 min and blocked for 2 h at 4 °C in bovine serum albumin (BSA, 5%, wt/vol.). The cells were then labeled overnight at 4 °C using polyclonal anti-MUC2 (1:100) and anti-NLRP6 (1:100), respectively, followed by three washings with phosphate-buffered solution–Tween-20 (PBST) for 10 min. Then, the cells were stained for 1 h using FITC-conjugated anti-rabbit (1:200) and Cy5-conjugated anti-rabbit (1:200) secondary antibodies. After washing thrice with PBST for 5 min, the cells were cultured with DAPI cell nuclear dye for 5 min and then washed thrice with PBST for 3 min. Lastly, the cells were washed and mounted on glass slides, and images were acquired using a fluorescence microscope (Olympus DP71, Tokyo, Japan).

### 2.7. Total RNA Extraction and Gene Expression Detection

Total RNA was extracted and then transcribed into cDNA (42 °C for 10 min, 65 °C for 10 s, stored at 4 °C) using the PrimeScript™ RT reagent Kit (TaKaRa, Osaka, Japan). The primers of *MUC2* (GenBank: NG_051929), *TFF3* (GenBank: NM_003226), *RELMB* (GenBank: NM_032579.3), *CHST5* (GenBank: NG_029853) and *GAL3ST2* (GenBank: NG_046977) are outlined in Table 1. The expressions of *MUC2*, *TFF3*, *RELMB*, *CHST5* and *GAL3ST2* genes were detected using reverse transcription-polymerase chain reaction (qRT-PCR), with GADPH as the reference gene. The reaction system was made to a total volume of 20 μL, comprising 1 μL template cDNA, 0.5 μL forward primer/reverse primer (10 μM), and 10 μL of TB Green^®^ Fast qPCR Mix. The data were calculated using the 2^−ΔΔCt^ method [19].

### 2.8. Statistical Analysis

All data were analyzed using One-Way ANOVA multiple comparisons to assess differences between the groups and were expressed as mean ± SEM. *p* values < 0.05 were considered statistically significant. GraphPad Prism v9.0 (GraphPad Software company, San Diego, California, USA) was applied to draw bar charts of the above data.

## 3. Results

### 3.1. Effects of the Four Chemicals on Cell Viability of LS174T Cells

**Figure 1:**
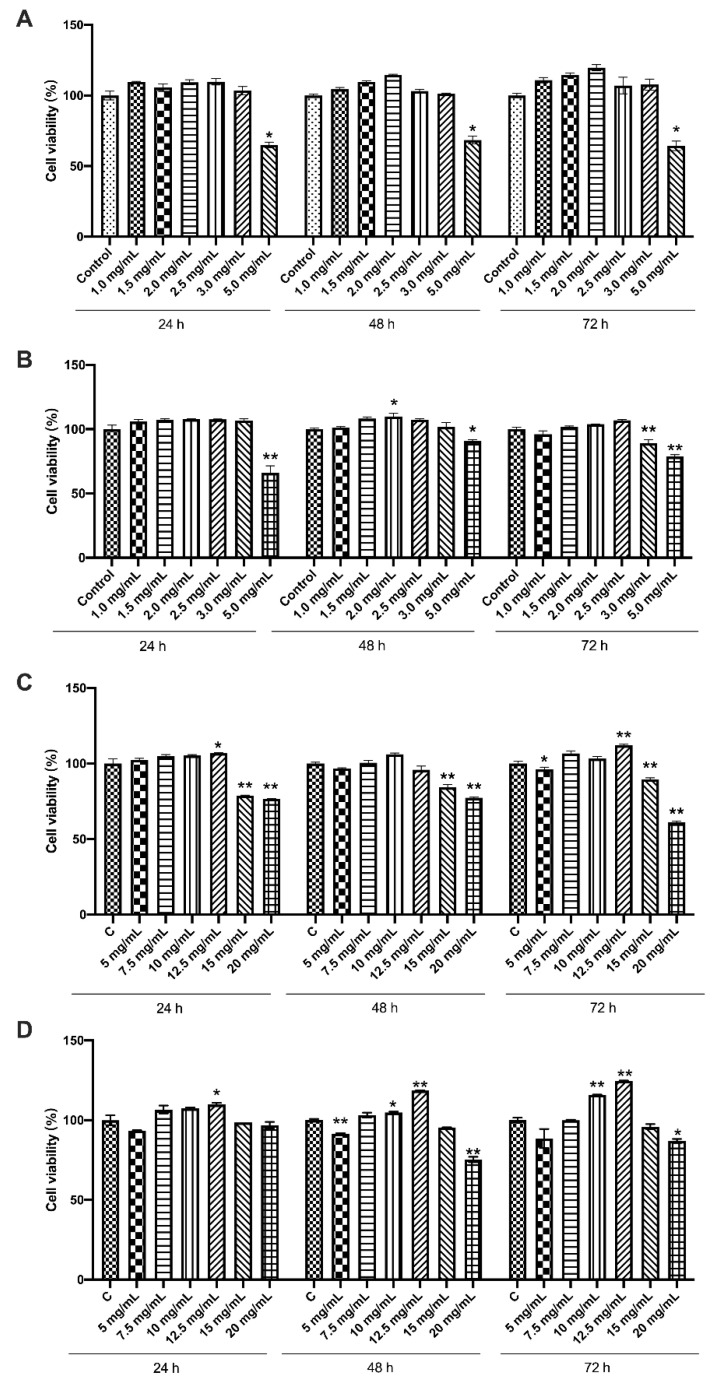
The cell viability and growth status of LS174T cells with or without prebiotic treatment. (**A**) 2′-FL; (**B**) 3′-SL; (**C**) GOS; (**D**) Lac. Significance was determined using one-way ANOVA and expressed as mean ± SEM. (*n* = 5 for each group). * *p* < 0.05; ** *p* < 0.01 when compared with control group.

Based on the previous studies [16,17] and their actual concentrations in milk, we selected a relatively wide range of the four prebiotics to treat LS174T cells. As shown in Figure 1, compared with the control group, no significant changes were observed in the cell viability of LS174T cells treated with 1.0–2.5 mg/mL 2′-FL and 3′-SL. Moreover, 1.5, 2.0 and 2.5 mg/mL 2′-FL could slightly enhance cell viability when cultured for 72 h. On the other hand, the cells treated with 5.0 mg/mL 2′-FL and 3′-SL exhibited a significantly lower viability (*p* < 0.05). The viability of cells in the 15 and 20 mg/mL GOS or Lac groups were significantly decreased compared with the control group (*p* < 0.05). Cell viability at 24 and 48 h showed a similar tendency to that observed at 72 h. Therefore, the doses 1.5, 2.0 and 2.5 mg/mL of 2′-FL and 3′-SL, and 7.5, 10 and 12.5 mg/mL of GOS and Lac were selected for subsequent experiment to determine the optimal dosage.

### 3.2. Effects of the Four Chemicals on Mucin Secretion of LS174T Cells under Normal Condition

**Figure 2:**
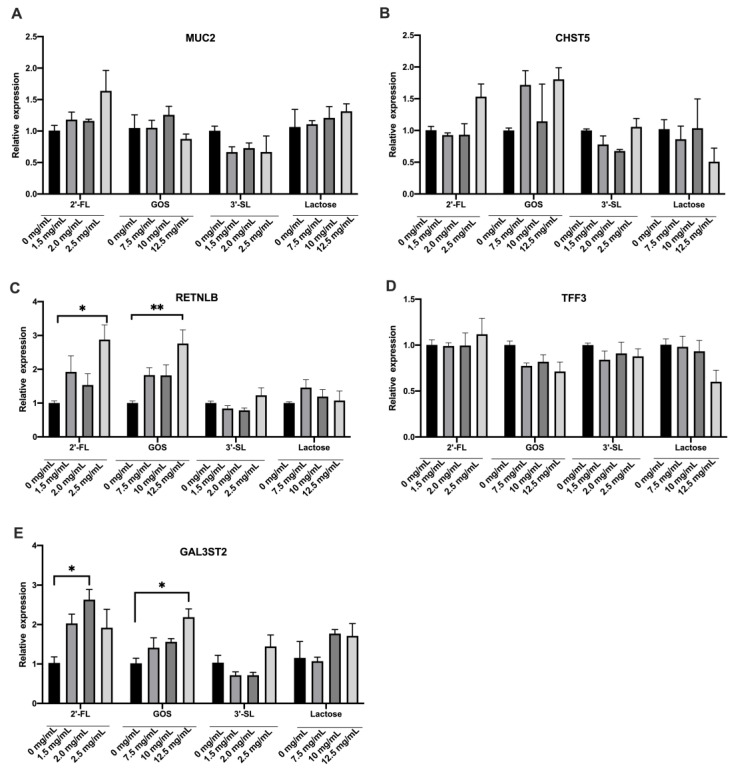
The mRNA expression levels of mucin secretion-related genes under steady-state condition. Significance was determined using one-way ANOVA and expressed as mean ± SEM. (*n* = 5 for each group). * *p* < 0.05. ** *p* < 0.01 when compared with the control group. (**A**–**E**), the mRNA expression of *MUC2*, *CHST5*, *RETNLB*, *TFF3* and *GAL3ST2* genes.

To further explore the effects of the different prebiotics on the mucin secretion ability of LS174T cells under steady-state conditions, the cells were treated with 1.5, 2.0 and 2.5 mg/mL of 2′-FL and/or 3′-SL, and 7.5, 10 and 12.5 mg/mL of GOS and/or Lac. After incubation for 72 h, the change in expression of mucin secretion-related genes was assessed. As shown in Figure 2, there was no significant difference in MUC2 gene expression among the different treatment groups (*p >* 0.05). The expression of *RETNLB* gene in 2.5 mg/mL 2′-FL and/or 12.5 mg/mL GOS, as well as of *GAL3ST5* in 2.0 mg/mL 2′-FL and 12.5 mg/mL GOS were significantly up-regulated compared with the control group (*p* < 0.05). The 3′-SL and Lac showed no obvious influence on mucin secretion in a homeostasis condition. Based on these results, the doses of 2.5 mg/mL 2′-FL and 3′-SL, and 12.5 mg/mL GOS and Lac were chosen for the following experiments.

### 3.3. Effects of the Four Chemicals on MUC2-Related Gene Expression under an Inflammatory Condition

**Figure 3:**
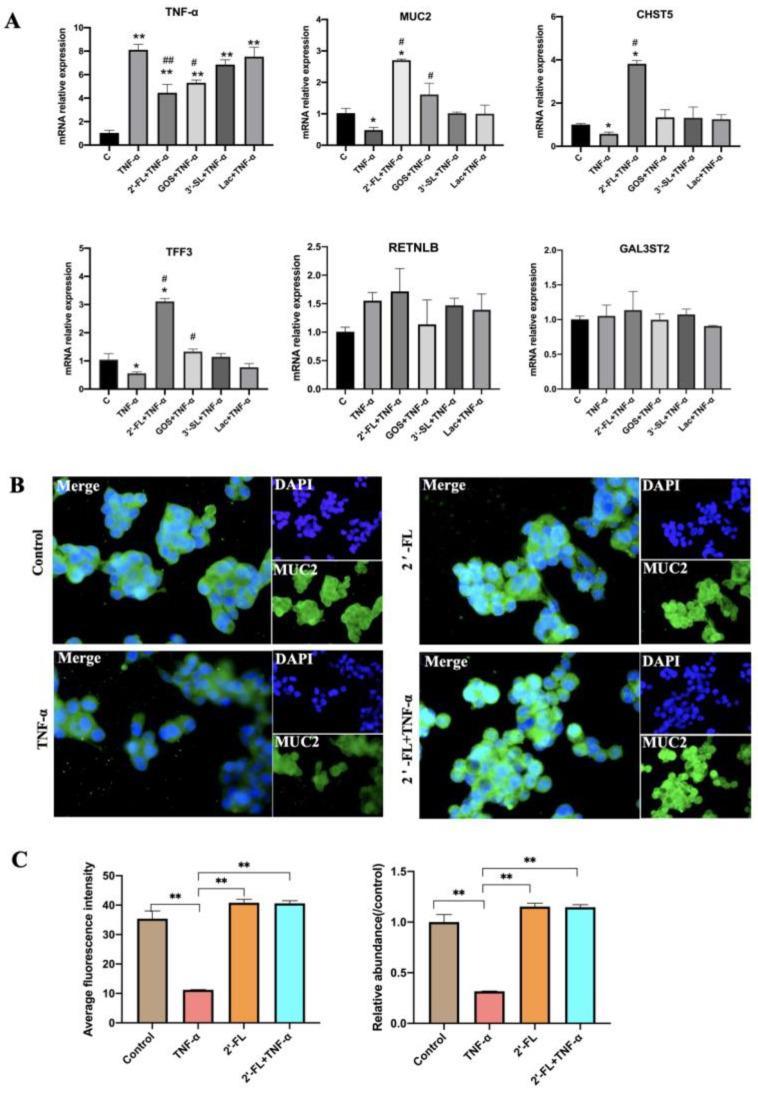
The mRNA expression levels of mucin secretion-related genes and cell immunofluorescence staining of LS174T cells under an inflammation condition. (**A**) Mucin secretion-related genes; (**B**) Cell immunofluorescence staining; (**C**) Average fluorescence intensity of MUC2 and its relative abundance (compared to control group), calculated using Image J pro. Significance was calculated using One-Way ANOVA and expressed as mean ± SEM. (*n* = 5 for each group). * *p* < 0.05. ** *p* < 0.01 compared to the control group; ^#^ *p* < 0.05. ^##^ *p* < 0.01 compared to the TNF-α group.

To construct the inflammatory model, 10 ng/mL TNF-α was used to treat LS174T cells. As shown in Figure 3, TNF-α induced a sharp increase in the mRNA expression of TNF-α when compared with the control group (*p* < 0.05; *p* < 0.01), which could be inhibited by 2′-FL and GOS. In regard to the mucin secretion indexes, the gene expression of *MUC2*, *CHST5* and *TFF3* was down-regulated in the TNF-α group compared with control group (*p* < 0.05). However, in the 2′-FL + TNF-α group, these genes were significantly up-regulated when compared with TNF-α group (*p* < 0.01). GOS also showed the ability to enhance the expression of *MUC2* and *TFF3* (*p* < 0.05). The ability of 2′-FL was 1.68 times higher than that observed for GOS. 3′-SL and Lac had no obvious effect on the gene expression of *MUC2* and mucin secretion related genes. Cell immunofluorescence staining confirmed that 2′-FL enhanced the expression of MUC2 protein under inflammatory state/condition.

### 3.4. 2′-FL Enhanced MUC2 Secretion in DSS-Induced Colitis Mice

**Figure 4:**
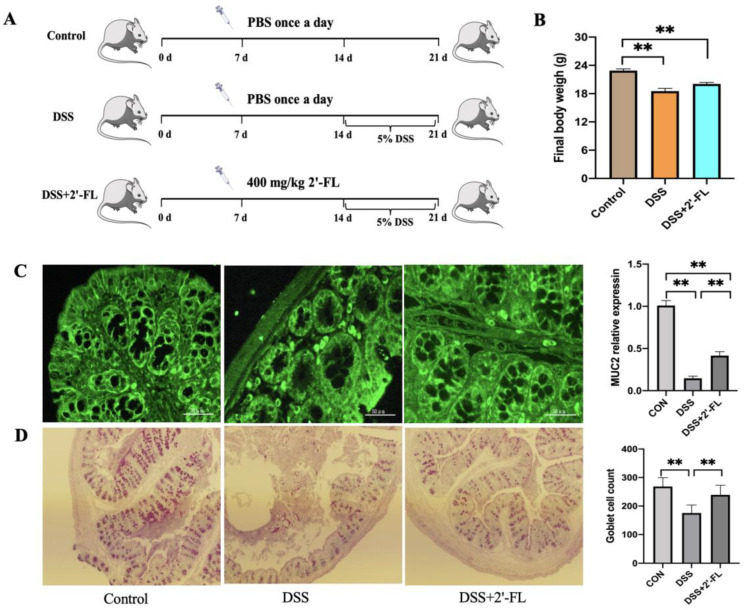
2′-FL enhanced MUC2 secretion in DSS-induced colitis mice. (**A**) The schedule of chemical treatment. (**B**) The final body weight of mice. (**C**) Representative fluorescent images and statistical analysis of MUC2. (**D**) PAS-stained distal colon sections showing goblet cells in colon tissue and its statistical analysis. Significance was determined using One-Way ANOVA and expressed as mean ± SEM. (*n* = 6 for each group). ** *p* < 0.01.

Here, the colitis model, a widely acknowledged inflammatory model, was used to evaluate the potential of 2′-FL in inhibiting inflammation in vivo. The results showed that, after treatment with DSS for 7 days, the MUC2 expression and goblet cell numbers in the DSS group were significantly lower compared to the control group, while the DSS + 2′-FL group exhibited a higher level of MUC2 and more goblet cells than the DSS group (Figure 4).

### 3.5. NLRP6 Is Necessary for MUC2 Secretion

**Figure 5:**
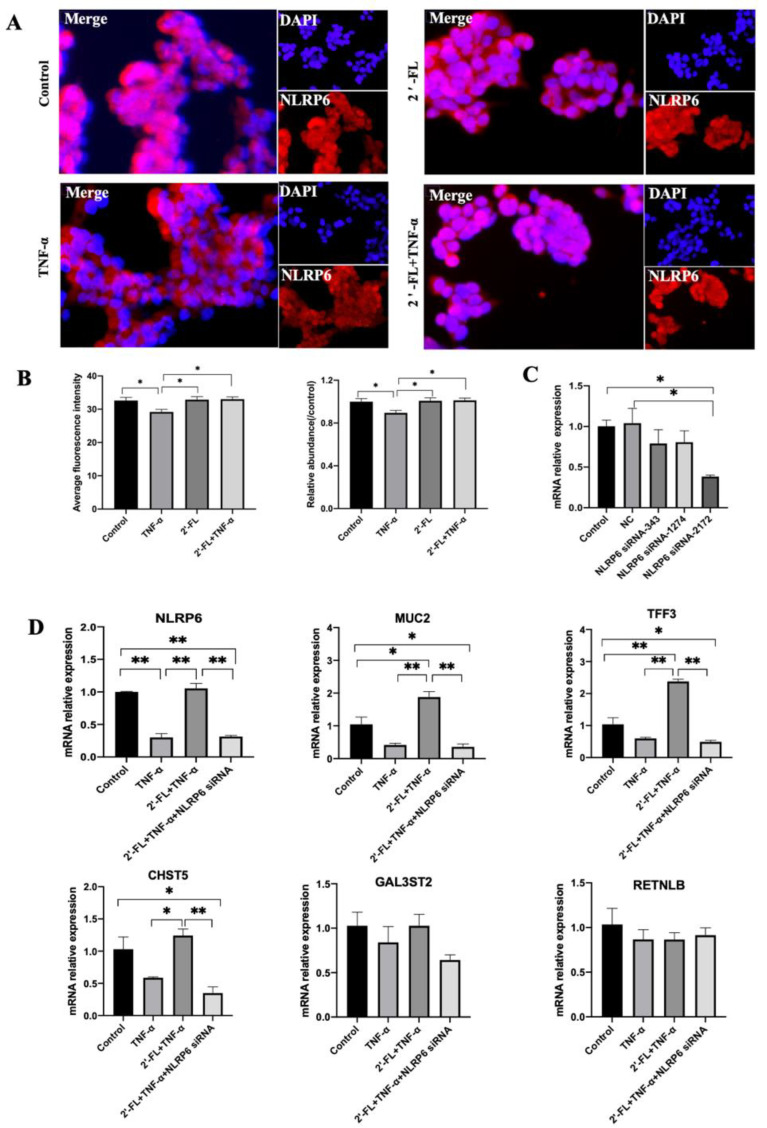
NLRP6 and expression of mucin-related genes measured before and after *NLRP6* gene silencing. (**A**) 2′-FL exposure (2.5 mg/mL) for 72 h increased NLRP6 expression in LS174T cells (representative images). (**B**) Statistics analysis of average of fluorescence intensity and its relative abundance (compared to control group) using GraphPad Prism v9.0. (**C**) The screening of *NLRP6* siRNA primers. (**D**) The mRNA expression levels of mucin secretion-related genes after *NLRP6* gene silencing. Significance was determined using One-Way ANOVA and expressed as mean ± SEM. (*n* = 5 for each group). * *p* < 0.05. ** *p* < 0.01 compared to the control group.

NLRP6 is reported to regulate MUC2 secretion in goblet cells [20] and its crucial role was demonstrated in a previous study via the enhancement of MUC2 expression in a mouse colitis model [15]. To further validate the roles of NLRP6 in an in vitro inflammatory model, cell immunofluorescence staining was performed in this study. The results showed that, after TNF-α stimulation, *NLRP6* expression was significantly decreased compared to the control group (*p* < 0.05), based on the decreased average fluorescence intensity (Figure 5A,B). However, 2′-FL treatment significantly improved its expression (*p* < 0.05).

We further performed an siRNA gene experiment to test the role of *NLRP6*. After siRNA-2172 treatment, the expression of *NLRP6* significantly decreased compared to the control group (Figure 5C, *p* < 0.05). However, there were no obvious differences in *NLRP6* expression between siRNA-343 and siRNA-1274 treatment groups and the control group (Figure 5C, *p* > 0.05), suggesting that these two sequences failed to silence the gene. Therefore, in the following experiment, we adopted siRNA-2172 to silence *NLRP6*. Compared to the 2′-FL + TNF-α group, the MUC2 and its related two genes (*TFF3* and *CHST5*) were all significantly decreased in the 2′-FL + TNF-α + NLRP6 siRNA group (Figure 5D, *p* < 0.05). This suggests that 2′-FL was unable to improve the *MUC2* secretion under inflammatory condition after *NLRP6* gene silencing, demonstrating that *NLRP6* is essential for *MUC2* expression in goblet cells.

### 3.6. 2′-FL Suppression of TNF-Induced Inflammation via Regulating the TLR4/MyD88/NF-κB Pathway

**Figure 6:**
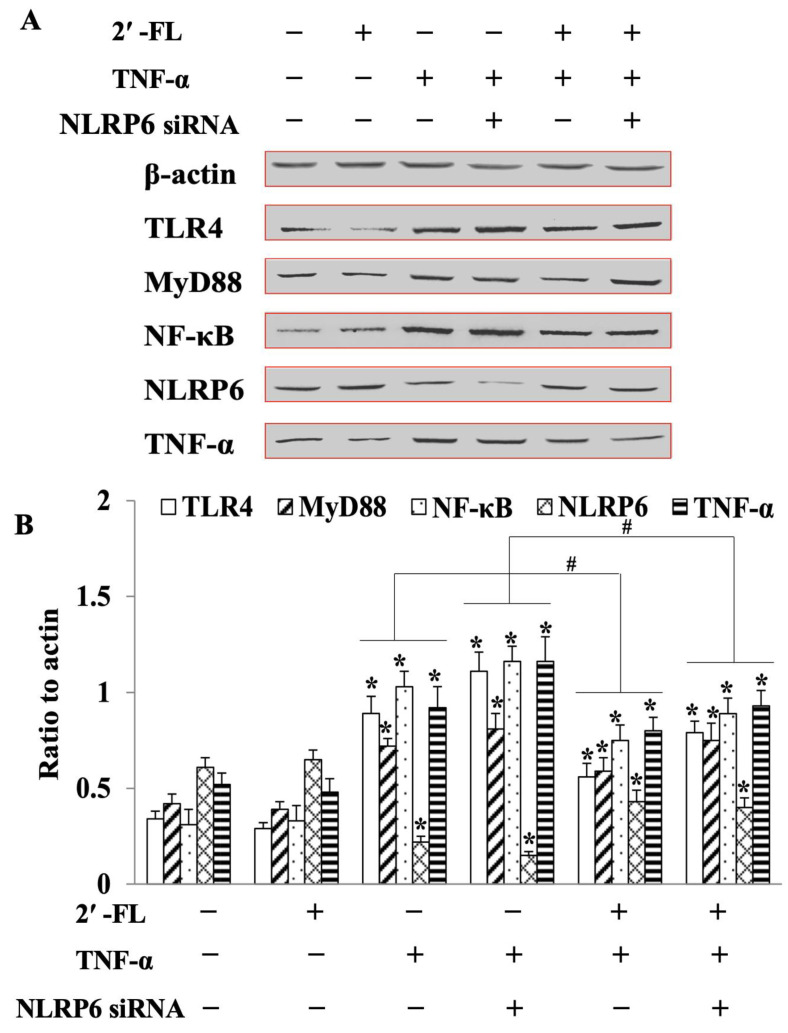
Protein expression of cytokines in the TLR4/MyD88/NF-κB pathway in LS174T cells. (**A**) Western blotting bands of Actin, TLR4, MyD88, NF-κB and NLRP6 in cells. Actin was regarded as the internal reference. (**B**) Densitometric quantitation for normalized proteins relative to Actin, analyzed using Image J software. Significance was determined using One-Way ANOVA and expressed as mean ± SEM. (three replicated tests). * *p* < 0.05 compared with the control, ^#^ *p* < 0.05 compared with the TNF-α group.

As shown in Figure 6, in the NLRP6 siRNA group, the expression of TLR4, MyD88, NF-κB, and TNF-α levels were remarkably increased compared to the control (*p* < 0.05). However, opposite results were observed in the 2′-FL + TNF-α group with a sharp decrease in the expressions of toll-like receptor 4 (TLR4), myeloid differential protein-88 (MyD88), nuclear factor kappa-B (NF-κB) and increased levels of NLRP6 (*p* < 0.05), indicating that NLRP6 could be upstream of the TLR4-related pathway. More importantly, after NLRP6 silencing, 2′-FL was still able to decrease the levels of the above inflammatory factors, suggesting that, in goblet cells, 2′-FL could not only promote MUC2 secretion, but also suppress inflammatory cytokine expression, to reduce inflammation. Furthermore, this last effect was independent, at least in part, of the first one.

## 4. Discussion

HMOs cannot be digested and absorbed in the small intestine [21], but they can be fermented and utilized by gut microbiota in the colon to improve intestinal health [22]. In the mammary gland, HMOs are formed by adding monosaccharides to a lactose core via specific glycosyltransferases, and then further decorated by fucose and sialic acid. Based on their structure, HMOs can be classified as fucosylated HMOs (2′-FL as a representative), sialic acid HMOs (3′-SL as a representative) and non-fucosylated neutral HMOs [23]. Sialic acid and fucose in the terminal part prevent HMOs from being utilized by gut bacteria that do not possess the enzymes (glycosyl hydrolases) to break down these monosaccharides [24]. Similar to HMOs, GOS consists of mostly galactose and a little glucose, which contains no further decoration. The content of HMOs is relatively low in milk, and the development of its in vitro synthesis is slow due to the restriction of biotechnology. Therefore, for a long time, GOS was used as a supplement in infant formula to mimic the function of HMOs. However, the structural discrepancy between the two is the basis for the difference in their biological functions, which urges further exploration.

In this present study, we evaluated the ability of 2′-FL, 3′-SL and GOS to enhance mucin secretion under two different cellular environments, i.e., homeostasis and inflammatory environment. Most previous studies reported that HMOs increased MUC2 expression in in vivo inflammatory models [25]. However, the roles of HMOs under a steady-state situation remain unclear. Here, we found that 2′-FL, 3′-SL, GOS and Lac exhibited low ability to facilitate mucin secretion in the homeostasis cellular environment. In cοntrast to this, 2′-FL and GOS showed an obvious effect in improving mucin barrier after TNF-α treatment, and the ability of the former was higher than the latter, where *MUC2* expression was improved by 5.64 and 3.36 fold compared to the TNF-α group, respectively. Cheng et al. [18] reported that 2′-FL improved the MUC2 expression of LS174T cells exposed to an IL-13 environment. In our present study, the expression of *TFF3* and *CHST5* in the 2′-FL + TNF-α and GOS + TNF-α groups were enhanced by 3.82 and 1.43 times compared to the control group, and by 6.67 and 2.33 fold compared to the TNF-α group, respectively. TFF3 is of significant importance for the mucin barrier, not only for protecting mucus integrity, but also for increasing the expression of tight-junction proteins, such as claudin-1 and Zo-1 [26]. However, those functions are not observed in steady-state, suggesting that there might be chemical signals such as TLRs, to activate 2′-FL and GOS to exert their function in the inflammatory state.

Both 3′-SL and Lac had little influence on MUC2 expression either in steady situation or under the TNF-α challenge. Currently, 3′-SL is known to improve cognitive ability [27] and anti-inflammatory effects in various parts of the body [28,29,30], indicating that 3′-SL is mainly absorbed into the blood circulation and then arrives at the brain or other parts of the body to exert its functions of mediating neuronal communication and alleviating inflammation. Lac is highly present in milk and is also the core structure of HMOs and GOS. It is an undesirable component for Lac-intolerant consumers. However, lactase can hydrolyze Lac into monosaccharides, allowing the non-hydrolyzed Lac to reach the colon to be further fermented by microbiota and colonize, further exerting health benefits. Cederlund et al. [31] reported that Lac improved the expression of cathelicidins antimicrobial peptide (CAMP) by activating the MAPK pathway in HT29, Caco-2, and T84 cell lines. In our study, Lac showed little relationship with mucin secretion in vitro. Figueroa- Figueroa-Lozano et al. [32] demonstrated that Lac mixed with GOS improved the expression of genes related to mucus in goblet cells, which, however, might be the effect of GOS.

NLRP6 belongs to the NLR subfamily, which is composed of NLRP6, caspases and ASC. There is evidence showing that, depending on the type of cell, NLRP6 can exert different roles in regulating inflammation [33]. Through the assembly of an inflammasome, NLRP6 was shown to prevent dextran sodium sulfate (DSS)-induced colitis by regulating IL-18 levels in colonic epithelial cells [34]. NLRP6 prevented the development of alcoholic hepatitis by blocking the NF-κB signaling pathway in hepatic stellate cells [35]. In our previous study [15], NLRP6 was found to be involved in the process of 2′-FL alleviating DSS-induced colitis and promoting MUC2 expression in C57BL/6J mice. Thus, this study explored the interactions between MUC2 and 2′-FL in enhancing mucin expression in goblet cells (LS174T cells). Our results showed that, compared to the 2′-FL + TNF-α group, MUC2 and its related two genes (TFF3 and CHST5) were all significantly decreased after *NLRP6*-siRNA treatment. Interestingly, the expression of TNF-α, IL-6 and NF-κβ were all suppressed in the 2′-FL + TNF-α + NLRP6-siRNA group compared to the TNF-α group, indicating that the anti-inflammatory effect of 2′-FL could be independent of NLRP6, although the latter was essential for MUC2 expression in goblet cells. At the protein level, it was found that in the TNF-α +NLRP6 siRNA group, TLR4, MyD88, NF-κB, and TNF-α levels were significantly up-regulated compared to the control group, which was reversed in the 2′-FL + TNF-α group. After *NLRP6* was silenced, 2′-FL could still decrease the levels of the above inflammatory factors, confirming the function of 2′-FL in goblet cells via multiple processes, i.e., promoting mucin secretion through NLRP6 and suppressing inflammation through inhibiting the TLR4-related pathway. These results are consistent with the study of Sodhi et al. [36], who found that 2′-FL docked into the binding pocket of the complex of TLR4-myeloid differentiation factor 2 (MD2) complex to inhibit TLR4 signaling, which consequently alleviated necrotizing enterocolitis (NEC) in a mice model. In the cytoplasm, the signal caused by the 2′-FL dissolution of the MyD88 adapter via TLR4 was then transmitted to the nucleus via the NF-κB signaling pathway to regulate the process of inflammation.

## 5. Conclusions

In summary, 2′-FL and GOS, but not 3′-SL and Lac, were shown to increase the mRNA expression of *MUC2*, *TFF3* and *CHST5* only in an inflammatory state. Besides, 2′-FL exerted its function in goblet cells via multiple processes, such as promoting mucin secretion through NLRP6 and suppressing TLR4 related inflammation. These findings open a new venue to create more effective formulations to enhance intestinal health.

## Figures and Tables

**Table 1:** The primers of targeted genes.

Genes	Forward Primer Sequence (5′→3′)	Reverse Primer Sequence (5′→3′)
*CHST5*	CCC AGT GAG GAA CTG GTC TTC	ATC TGT GTT CCA GGA AAG CC
*GAL3ST2*	TGG GCG GCT TGC AGA GAT A	GCT CTA AGT CCG AGT GCA GGA
*MUC2*	AAC ACA GTC CTG GTG GAA GG	CAT TGT CAG GTC CCA CAC AG
*RETNLB*	CAC CCA GGA GCT CAG AGA TCT AA	ACG GCC CCA TCC TGT ACA
*TFF3*	CAT GTC ACC CCC AAG GAG TG	AGG TGC ATT CTG CTT CCT GC
*GADPH*	AAG ATC ATC AGC AAT GCC TCC TGC	ATG GAC TGT GGT CAT GAG TCC TTC
*NLRP6*-1374	GCA GUU UGC CGA GAA GGA ATT	UUC CUU CUC GGC AAA CUG CTT
*NLRP6*-2142	CCU UCU UCA UCC ACU CUU UTT	AAA GAG UGG AUG AAG AAG GTT
*NLRP6*-343	GUG UCC GAG UAC AAG AAG ATT	UCU UCU UGU ACU CGG ACA CTT

## Data Availability

Data is contained within the article.
